# The Bead Assay for Biofilms: A Quick, Easy and Robust Method for Testing Disinfectants

**DOI:** 10.1371/journal.pone.0157663

**Published:** 2016-06-17

**Authors:** Katharina Konrat, Ingeborg Schwebke, Michael Laue, Christin Dittmann, Katja Levin, Ricarda Andrich, Mardjan Arvand, Christoph Schaudinn

**Affiliations:** 1 Division for applied Infection Control and Hospital Hygiene, Robert Koch Institute, Berlin, Germany; 2 Advanced Light and Electron Microscopy, Robert Koch Institute, Berlin, Germany; Ghent University, BELGIUM

## Abstract

Bacteria live primarily in microbial communities (biofilms), where they exhibit considerably higher biocide tolerance than their planktonic counterparts. Current standardized efficacy testing protocols of disinfectants, however, employ predominantly planktonic bacteria. In order to test the efficacy of biocides on biofilms in a standardized manner, a new assay was developed and optimized for easy-handling, quickness, low running costs, and above all—repeatability. In this assay, 5 mm glass- or polytetrafluoroethylene beads in 24 well microtiter plates served as substrate for *Pseudomonas aeruginosa* biofilms. After optimizing result-relevant steps, the actual performance of the assay was explored by treating *P*. *aeruginosa* biofilms with glutaraldehyde, isopropanol, or peracetic acid in predefined concentrations. The aspired 5 log_10_ reduction in CFU counts was achieved by glutaraldehyde at 5% (30 min), and by peracetic acid at 0.3% (10 min). In contrast, 80% isopropanol (30 min) failed to meet the reduction goal. However, the main accomplishment of this study was to unveil the potential of the array itself; most noteworthy here, a reliable repeatability of the results. The new bead assay for biofilms is a robust, quick and cost-effective method for assessing the efficacy of biocides against biofilms.

## Introduction

The majority of bacteria live obviously in slime-encased microbial communities, which have been referred to as biofilms [1]. During their three billion years of evolution bacteria in biofilms have developed remarkable protection strategies against a wide range of biocides, while their planktonic counterparts remained comparatively susceptible [2]. The higher tolerance of biofilm-associated bacteria against biocides represents a serious problem in health care facilities or industrial production plants. In order to prevent bacteria from colonizing surfaces, for instance in a hospital, a wide variety of disinfections protocols are commonly applied [3]. The efficacy of disinfectants is tested using standardized methods, which are—up to now—predominantly based on planktonic bacteria [4]. Consequently, they do not take into account the elevated biocide tolerance of bacteria in biofilms [5]. If one is to assess the effectiveness of a disinfectant against biofilms with all required triplicates and repeats of different contact times and concentrations, microtiter plate based methods are best suited to handle larger numbers of samples [5]. The MBEC-assay (MBEC = minimum biofilm eradication concentration) of Innovotec Inc. (Edmonton, AB, Canada) is the only ASTM International (ASTM = American Society for Testing and Materials) approved microtiter plate based system so far and has been manifold employed to test biocides against bacterial biofilms [4, 6, 7]. The 96 testing positions of a MBEC plate make this system to a powerful high throughput device [8, 9]. However, many situations require the assessment of only a few parameters for which, nevertheless, an entire MBEC plate has to be sacrificed. The also ASTM approved CDC biofilm reactor (available at Biosurface Technologies Corporation, Bozeman, MT, USA), on the other hand, offers a maximum of 24 testing positions [10]. Its limitations are the necessity of a pumping system, a 16 liter media throughput per run, and the fact that the CDC biofilm reactor allows only one species to be cultivated at a time. An easy adjustment to sample size beyond the 24 testing positions by multiplying the setup is difficult for such a system.

These limitations generated a desire for a more flexible system that would allow for testing larger as well as small sample numbers. The aim of the present study was to develop a flexible, quick, easy andreliable assay to test the efficacy of biocides on bacterial biofilms at low costs.

## Material and Methods

(The entire bead assay protocol is available as flow chart in S1 Flow Chart).

### Bead Size and Material

3 and 5 mm glass beads served as substrate for the biofilm (Merck KGaA, Darmstadt, Germany) as well as 5 mm polytetrafluoroethylene (PTFE) beads (Hoch Kugelfertigung, Hassfurt, Germany). All beads were used only once.

### Biofilm Cultivation

Prior to their use, all beads were rinsed in a soap solution, washed in ddH_2_O, incubated overnight in 80% isopropanol, and finally thoroughly cleaned in ddH_2_O. The beads were then autoclaved and placed in the wells of a 24-well microplate (one bead per well). A tryptic soy broth (TSB) overnight culture of *Pseudomonas aeruginosa* (ATCC 15442) was diluted in TSB to approx. 1x10^5^ bacteria mL^-1^ and dispensed into the bead-containing 24 well microplate (1 mL per well). The microplate was then placed in a moisture chamber (Emsa clip and close storage container, Emsa, GmbH, Emsdetten, Germany) and incubated at 37°C for 24 h at 150 rpm on an orbital shaker.

### Biofilm Processing

After biofilm cultivation, each bead was dipped (using sterilized tweezers) in slow-motion in 2 mL sterile ddH_2_O (24 well microplate, one bead per well) to remove loosely attached bacteria and placed in a 2 mL microcentrifuge tube (due to their slightly bigger size recommended: 2.0 mL Safe Lock Tubes, Eppendorf) containing 2 mL phosphate buffered saline (PBS, 0.1 M, pH 7). Subsequently, the microcentrifuge tubes were sonicated in an ultrasonic bath (BactoSonic^®^, Bandelin, Berlin, Germany) at 40 kHz for 10 min using 200 W_eff_ to detach the biofilm from the bead surface and individualize the bacteria. 200 μL of each sample was transferred to the ‘A’ row wells of 96 well microplates. (Alternatively to standard 96-microplates, Eppendorf^™^ deepwell^™^ Plates 96 can be used with adjusted volumina). All samples were then diluted from row ‘A’ down to row ‘H’ in 1:10 steps (20 μL sample + 180 μL PBS) by using a multichannel pipette. 5 μL of each well was spot deposited with a multichannel pipette on a square tryptic soy agar plate (12x12 cm) (see S1 Spotting Grid). All plates were incubated overnight at 37°C and the CFU counted the next day. Only spotting areas with 5 to 50 CFUs were counted.

### Mixing and Bead Handling

All mixing steps involving microcentrifuge tubes were done on a vortex for 8 seconds (Vortex-Mixer Genie^®^ 2 Digital, Scientific Industries, Inc., Bohemia, NY, USA). For all dilution steps in microplates as well as for 5 μL spot-plating on square agar plates, the liquid volumes were mixed by pipetting up and down 20 times in combination with stirring using the pipette tips. For all bead-handling steps, sterilized tweezers with serrated, angled tips were used for a secure grip (DUMONT 24/25, Plano, Wetzlar, Germany). In order to grab a glass bead safely with the tweezers it is recommended to tilt the microplate to a 45 degree angle. In case a bead was not properly grabbed or the tweezers slipped the bead in question was discarded and a replacement used instead.

### Shaker Orbits Comparison

While one set of 5 mm glass beads was cultivated for 24 h on a shaker with an 8 mm orbit at 150 rpm, a second set was cultivated using a shaker with a 25 mm orbit at 150 rpm. The biofilm processing was done as described above.

### Repeatability Testing

*P*. *aeruginosa* biofilm was cultivated on 18 beads according to the standard protocol and processed as described above to determine the number of CFU per bead. This experiment was then repeated twice to achieve a total of three runs.

### Scanning Electron Microscopy (SEM)

The biofilm cultivation was performed as described above. A triplicate of 5 mm glass beads were washed by dipping in ddH_2_O and then fixed in a solution of 4% paraformaldehyde and 0.25% glutaraldehyde in 20 mM HEPES buffer for 48 h at 20°C. The biofilm of the second batch of glass bead triplicates was removed by sonication for 10 min as described before, then the bead was dip-washed in ddH_2_O and finally fixed in the before mentioned fixative. Subsequently, all samples were air-dried, mounted on a stub with adhesive carbon tape and Acheson silver (DAG1415M, Plano GmbH, Wetzlar, Germany), then sputter coated with a 12 nm layer of gold-palladium and examined in the SEM (ZEISS 1530 Gemini, Carl Zeiss Microscopy GmbH, Germany) operating at 3 kV using the in-lens electron detector.

### Live/Dead^®^ Imaging of Biofilms

After the cultivation of biofilms (see above), a 5 μL droplet of LIVE/DEAD^®^ BacLight^™^ (Thermo Fisher Scientific, Waltham, MA, USA) was placed in the center hole (2 mm diameter) of a 1x1 cm^2^ piece of a silicone foil (Bess Medizintechnik, Berlin, Germany) in an ‘Attofluor^®^ Cell Chamber’ (Thermo Fisher Scientific, Waltham, MA, USA). Subsequently, a biofilm covered glass bead was placed on the droplet so that the biofilm could be imaged using a confocal laser scanning microscope (LSM780, Carl Zeiss AG, Oberkochen, Germany) equipped with the Plan-Apochromat 20x/0.8 DIC objective.

### Live/Dead^®^ Imaging of Bacteria after Sonication

Biofilm cultivation was done as described above followed by 10 min sonication. 100 μL of this suspension was added to 0.5 μL of LIVE/DEAD^®^ stain, then spotted on a glass slide and imaged with the confocal laser scanning microscope (LSM780, Carl Zeiss AG, Oberkochen, Germany).

### Visualization of the Biofilm Matrix

The biofilm was cultivated as described in “biofilm cultivation”. Concanavalin A (binds to alpha-D-mannose and alpha-D-glucose, final concentration: 50 μg mL^-1^) were mixed with Syto60 (final concentration: 5 μM) and then the biofilm was stained as outlined in the staining protocol “LIVE/DEAD^®^ imaging of biofilms”.

### Image Processing

Images have been cropped, adjusted for optimal brightness and contrast (applied to the whole image) using Photoshop Lightroom^®^ (Adobe Systems, San Jose, CA, USA).

### Biofilm Disinfection Assay

The following disinfectants were tested:

A) Isopropanol (99.8%, p.a. Carl Roth GmbH& Co.KG, Karlsruhe, Germany) in concentrations of 10%, 25%, 50%, 70% and 80% for a contact time of 30 min. PBS (0.1M, pH7) served as neutralizer. B) Wofasteril (peracetic acid 40%; Kesla Pharma Wolfen GmbH, Bitterfeld-Wolfen, Germany) in concentrations of 0.01%, 0.05%, 0.1%, 0.2% and 0.3% peracetic acid for a contact time of 10 min. A solution of 0.5% sodium sulfite in PBS (0, 1M, pH 7) served as neutralizer. All solutions were freshly prepared. C) Glutaraldehyde (25%, Merck KGaA, Darmstadt, Germany) in concentrations of 0.001%, 0.02%, 0.1%, 0.5%, 1.0% and 5.0% for a contact time of 30 min. A solution of 1% glycine, 0.05% tween 80 in 0.1M PBS served as neutralizer. After biofilm cultivation, each bead was dipped in slow-motion in 2 mL sterile ddH_2_O (24 well microplate, one well per bead) and placed in a 2 mL microcentrifuge tube containing 200 μL disinfectant. After the incubation at 20°C, 1800 μL of the neutralizing agent were added to each microcentrifuge tube. Beginning with the sonication step the biofilms were processed as described under “biofilm processing”. All dilution steps were done in neutralizing agent. The controls were treated with PBS (0.1M, pH7) instead of the disinfectant.

### Treatment of Planktonic Bacteria

1 mL of a TSB overnight culture of *P*. *aeruginosa* (ATCC 15442) was added to 39 mL TSB and continued to cultivate at 37°C with 250 rpm to an optical density of 0.8. 20 μL of this culture was transferred to 2 mL Eppendorf tubes containing 180 μL glutaraldehyde (triplicates of: 0.001, 0.02, 0.1, 0.5, 1.0, 5.0%) as well as a triplicate of controls with PBS (0.1M, pH 7) and incubated for 30 min. From here on, the protocol “biofilm treatment with glutaraldehyde” was applied.

### Calculation of Reduction

All experiments were performed three times in triplicates. At first, all CFU counts were multiplied by the dilution factor. The spot-plated numbers were then multiplied with the factor 200, while the spread-plated counts were multiplied with 10 to achieve the desired reference volume of 1 mL. All results were logarithmized and the arithmetic mean of each experimental set was calculated. Whenever the CFU count was zero, the value “1” was assigned [11]. The efficacy of a disinfectant is represented by the reduction and calculated according to the following equation: log_10_R = log_10_ N_0_ –log_10_ N (log_10_ N_0_ = logarithmized CFU count of the untreated control; log_10_ N = logarithmized CFU count after disinfection). The arithmetic mean of the reduction was calculated for one set of experiments (three repeats in triplicates). The concentrations of disinfectants are deemed to be sufficiently bactericidal for disinfection if the mean reduction is a least five [12].

### Neutralizer Efficacy and Toxicity Test

In order to verify the efficacy of the respective neutralizing agent, 8.9 mL neutralizer was mixed with 1 mL of the highest used disinfectant concentration and incubated for 5 min at 20°C. This solution was then added to 0.1 mL *P*. *aeruginosa* suspension (1∙10^5^ bacteria mL^-1^) of sonicated biofilm from a glass bead and incubated for 30 min at 20°C. Serial dilution down to 10^−2^ was performed and 100 μL spread-plated on an agar plate. All neutralizing agent were freshly prepared.

In order to verify the absence of toxicity of the neutralizing agent, 9.9 mL neutralizer was added to 0.1 mL *P*. *aeruginosa* suspension (1∙10^5^ bacteria mL^-1^) of sonicated, dispersed biofilm from a glass bead and incubated for 30 min at 20°C. Serial dilution down to 10^−2^ was performed and 100 μL spread-plated on an agar plate.

As for the suspension control, 9.9 mL phosphate buffered saline (0.1M PBS, pH 7) was added to 0.1 mL *P*. *aeruginosa* suspension (1∙10^5^ bacteria mL^-1^) of sonicated, dispersed biofilm from a glass bead and incubated for 30 min at 20°C. Serial dilution down to 10^−2^ was performed and 100 μL spread-plated on an agar plate.

To demonstrate an adequate efficacy of the neutralizer in combination with absence of toxicity, the number of surviving bacteria should be equal or greater than 0.5 times the CFU counts of the *P*. *aeruginosa* suspension control (see above) [13].

### Statistical Analysis

In order to analyze the statistical significance of the repeatability, one-way analysis of variance (ANOVA) was performed. One requirement for the application of ANOVA is the homogeneity of observed variances, which was tested using the Bartlett-test. All calculations were done with Prism 5.0 GraphPad Software, Inc. La Jolla, CA, USA.

## Results

### Assay Standardization

**Fig 1:**
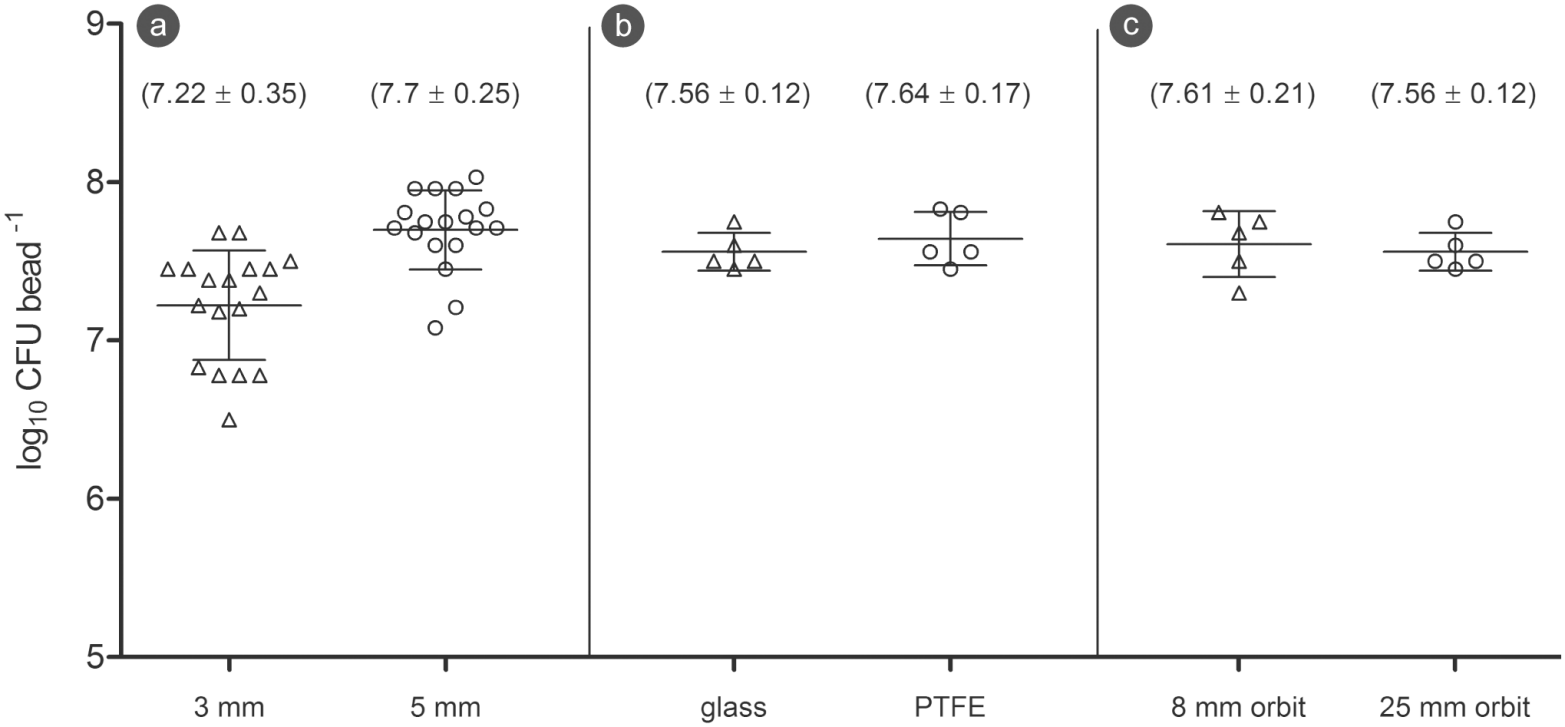
Assay standardization. (a) CFU counts of 3 and 5 mm glass beads. (b) CFU counts of 5 mm glass and polytetrafluoroethylene (PTFE) beads. (c) CFU counts of 5 mm glass beads cultivated on shakers with 8 and 25 mm orbit (The horizontal lines through the data points represent mean and standard deviation).

The first series of experiments served the purpose to identify the optimal conditions of the bead assay and to standardize a number of crucial steps. At first, 3 and 5 mm glass beads served as substrates in the bead assay to determine the number of adherent bacteria per bead, assess repeatability of the results, and test the handling properties of the different bead sizes. While the 3 mm glass beads showed in average 7.22 log_10_ CFU per bead on their surface, 5 mm glass beads yielded a slightly higher average of 7.70 7.7 log_10_ CFU per bead(Fig 1a). Due to the significantly better handling properties of 5 mm beads combined with marginally higher bacteria coverage, 5 mm beads were chosen for all further experiments.

In order to assess the influence of different materials on biofilm growth, two different bead materials were compared. The results clearly proved that the number of bacteria on glass beads and polytetrafluoroethylene beads were virtually equal (Fig 1b).

As the beads move around in their wells during the cultivation on the orbital shaker, the impact of the movement intensity was examined. Nevertheless, the CFU counts of the beads that were cultivated on a 25 mm orbit shaker coincided with those of the 8 mm orbit shaker (Fig 1c).

**Fig 2:**
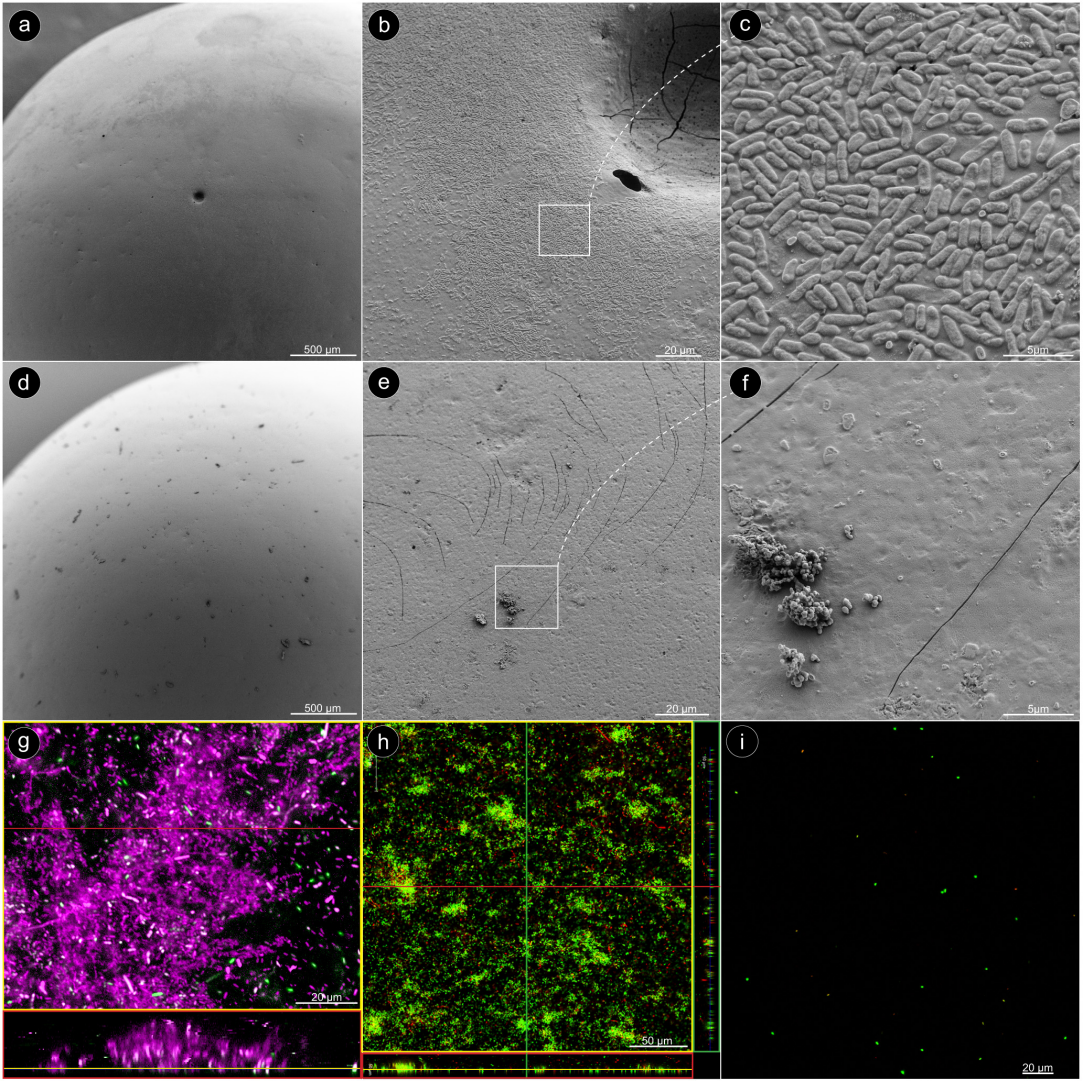
Microscopic characterization. (a) SEM image: overview on a glass bead after 24 h cultivation with *P*. *aeruginosa*. (b) The bead surface is evenly covered with biofilm. (c) The bacteria are densely arranged in a monolayer. (d) Overview on a glass bead after the biofilm had been removed by sonication. (e, f) The bead surface is virtually empty, except for some residual debris. (g) CLSM image: The sugar-matrix of the *P*. *aeruginosa* biofilm was stained with Concanavalin A (assigned color: magenta), and the bacteria with Syto60 (assigned color: green). (h) LIVE/DEAD^®^ staining of the biofilm on a glass bead. (i) LIVE/DEAD^®^ staining after the biofilm had been removed from the bead by sonication.

In order to ensure uniform biofilm coverage of the glass beads, a set of beads were imaged by scanning electron microscopy (SEM). All SEM images confirmed that the biofilms covered almost the entire glass beads in a uniform manner (Fig 2a–2c). After the sonication, however, virtually no bacteria were found on the surfaces of the beads (Fig 2d–2f), only some sporadic debris (Fig 2f). When the biofilm bacteria were stained with the DNA stain Syto60, and the biofilm matrix with Concanavalin A, almost the entire biofilm showed a strong Concanavalin A signal (assigned color: magenta) in the confocal laser scanning microscope (CLSM). Only few bacteria were completely free of sugar matrix (Fig 2g). After the glass beads were stained with the LIVE/DEAD^®^ assay, it became apparent that the bacteria in the biofilm were predominantly alive (Fig 2h). A crucial step in the bead assay was a complete detachment of the biofilm from the bead surface by sonication, and the dispersion of the biofilm to individual bacteria without affecting their vitality. Only then, the CFU counts truly reflect the number of bacteria in the biofilm. In order to ensure this fact, the detached bacteria were stained with LIVE/DEAD^®^ and imaged with the CLSM. The pictures confirmed that essentially all bacteria occurred as individual cells and most of them showed a green signal, which confirmed their vitality (Fig 2i).

**Fig 3:**
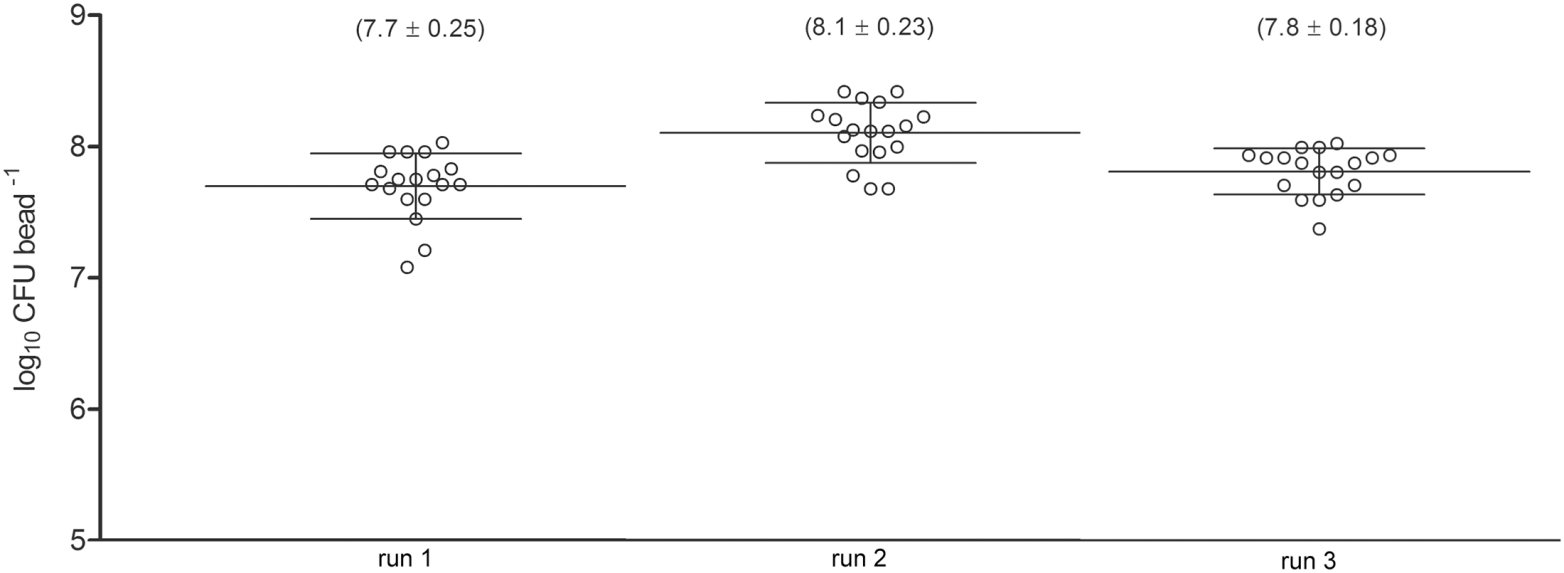
Testing for repeatability. CFU counts of three independent experiments with untreated biofilm on glass beads. (The horizontal lines through the data points represent mean and standard deviation).

Repeatability was regarded as one of the indispensable quality features for the bead assay, which was tested in three independent experiments of untreated control biofilms (Fig 3). The standard deviations of the three runs (18 beads per run) ranged between 0.18 and 0.25 and are equal (homogenous) across the runs according to the Barlett’s test (p = 0.3472). The one-way analysis of variance (ANOVA) showed no significant differences between different runs at a significance level of 0.05.

### Disinfectant Efficacy Testing Using the Bead Assay

For assessing the efficacy of disinfectants against *P*. *aeruginosa* biofilms, suitable concentrations were determined in pre-experiments to show a dose-dependent correlation.

**Fig 4:**
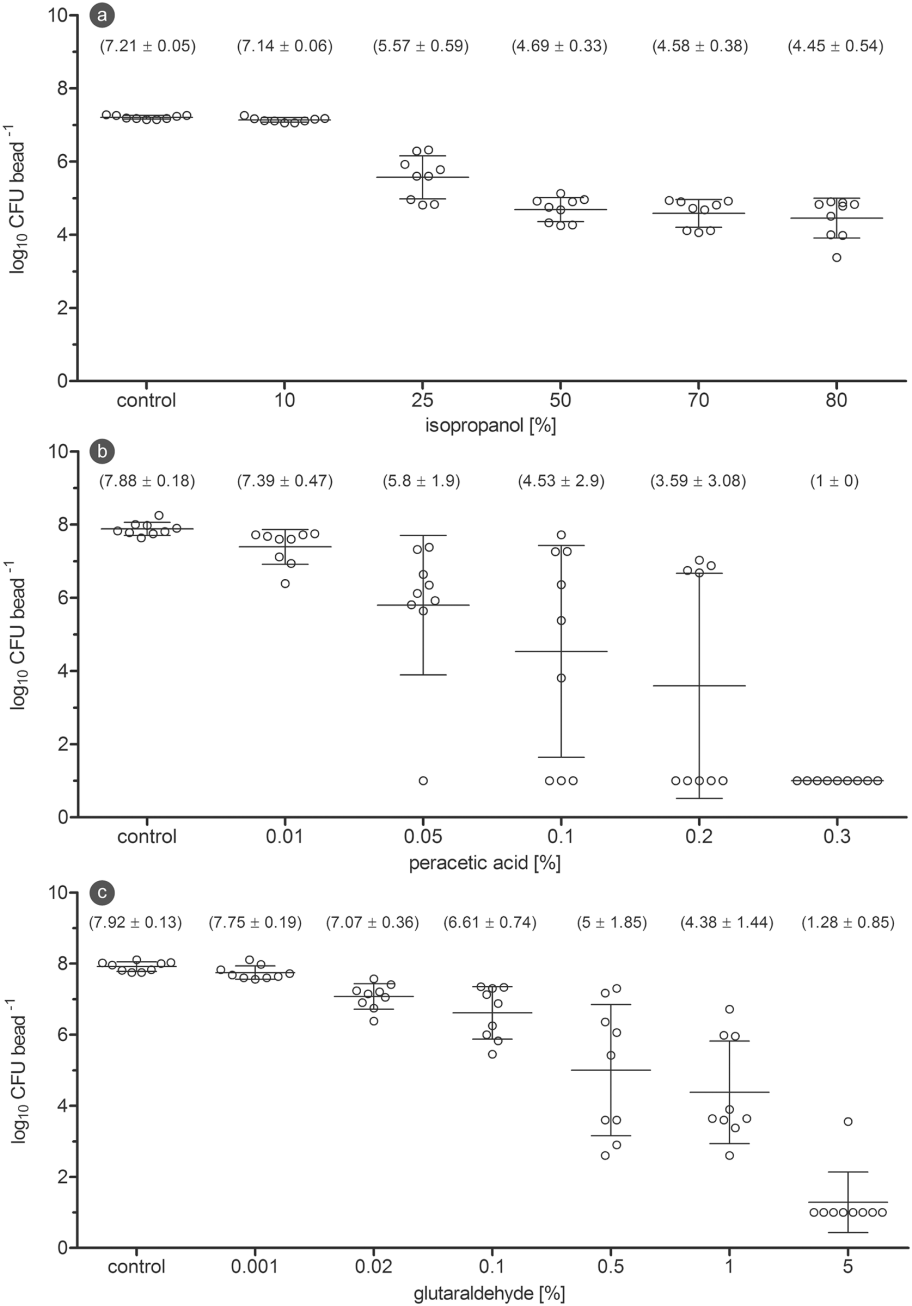
Disinfectant efficacy testing on biofilm. (a) CFU counts after 30 min isopropanol treatment of *P*. *aeruginosa* biofilm. (b) CFU counts after 10 min peracetic acid treatment of *P*. *aeruginosa* biofilm. (c) CFU counts after 30 min glutaraldehyde treatment of *P*. *aeruginosa* biofilm. (The horizontal lines through the data points represent mean and standard deviation).

**Fig 5:**
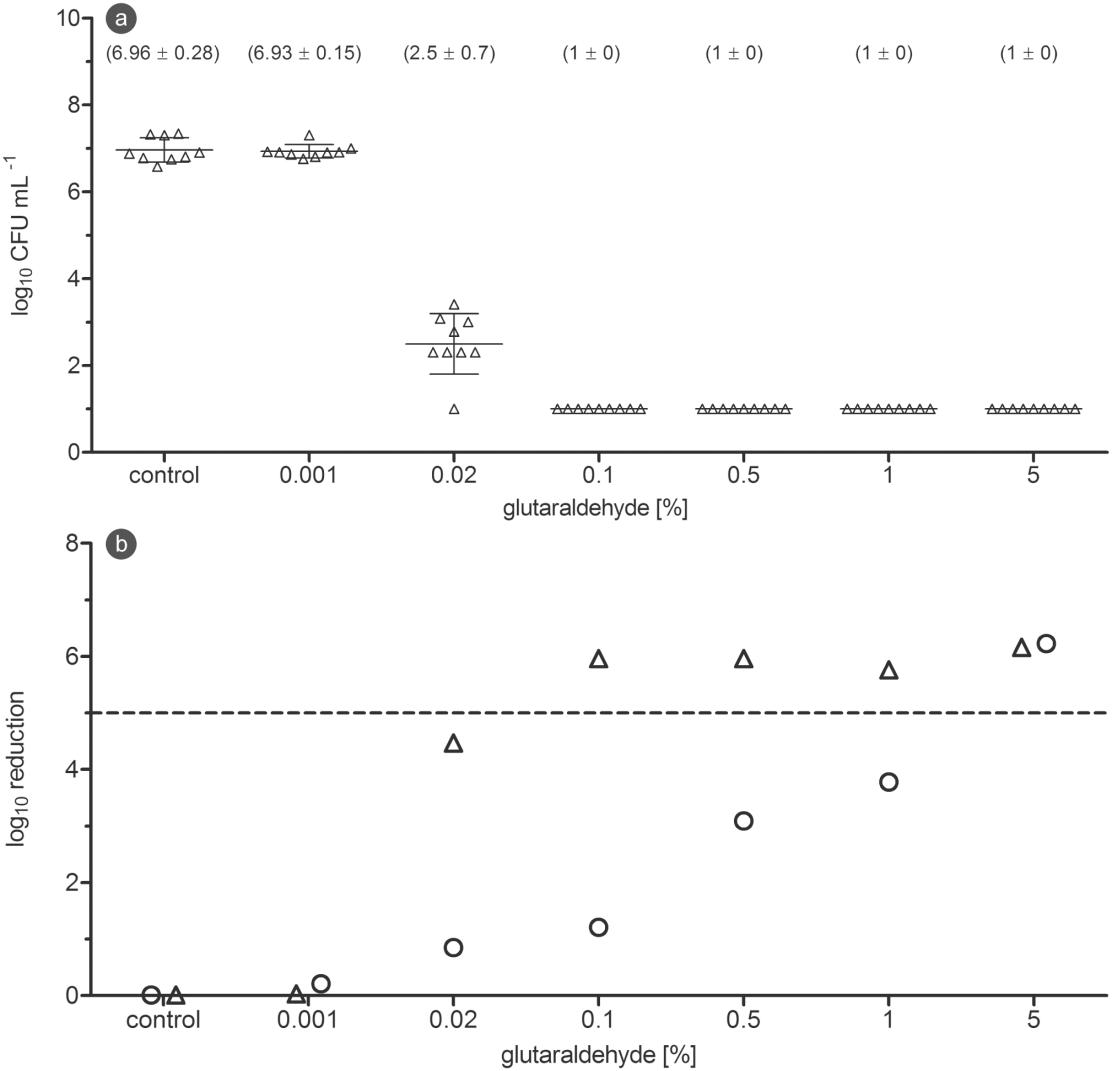
Reduction of planktonic bacteria and biofilm in comparison. (a) CFU counts after 30 min glutaraldehyde treatment of planktonic bacteria of *P*. *aeruginosa*. (b) Reduction after 30 min glutaraldehyde treatment of *P*. *aeruginosa* biofilm and planktonic bacteria (**O**: biofilm (mean of three independent experiments); **Δ**: planktonic (mean of three independent experiments); dashed line: 5log_10_ reduction goal).

The first disinfectant, isopropanol, was tested in concentrations between 10% and 80% against biofilm with a contact time of 30 min. While treatment with 10% isopropanol had no visible effect, the CFU counts started to decline at 25% and reached their lowest values at 80%. The 5 log_10_ reduction goal was not achieved (Fig 4a). Peracetic acid was tested in concentrations between 0.01% and 0.3% with a contact time of 10 minutes. Whereas 0.01% showed only a minor standard deviation, the CFU counts between 0.05 and 0.2% scattered widely. A complete inactivation was achieved at 0.3% (Fig 4b). When glutaraldehyde was tested in concentrations from 0.001% to 5% against the biofilm with a contact time of 30 min, the concentrations between 0.5 and 5% began to show a significant reduction in CFU counts and resulted in a reduction of over 5 log_10_ magnitudes (Fig 4c). After planktonic bacteria of the same strain were treated with the above mentioned glutaraldehyde concentrations, complete inactivation was already achieved at 0.1% (Fig 5a). The direct comparison of reduction values revealed that planktonic bacteria of *P*. *aeruginosa* were approximately 50-times more sensitive against glutaraldehyde, when compared to their counterparts in the biofilm (Fig 5b).

## Discussion

The aim of this study was to develop a new assay, which enables testing the efficacy of disinfectants on bacterial biofilms. The first challenge consisted of finding a reliable way to cultivate biofilms.

Glass as substrate to cultivate biofilms on is comparatively inexpensive and has been utilized since the early days of research on microbial communities [14]. Static (immobile) methods to cultivate biofilm—like for instance the widespread crystal violet biofilm assay [15]—are lacking a relative motion between biofilm and surrounding liquid media. The amount of shear forces, however, is considered to correlate with the rigidity of a biofilm [16]. A more rigid biofilm is supposed to be less susceptible to mechanical stress like washing steps, which will ultimately result in a more narrow deviation of data points. Rolling glass beads, on which biofilms form under a moderate amount of mechanical stress in a microplate, seemed to combine sufficient high flexibility with comparatively low running costs. Since microplates and microcentrifuge tubes as well as glass beads come in a great variety of sizes, up- and downscaling of the entire system is generally possible and would allow to adjusting the bead assay to a wide range of experimental requirements, if necessary.

One step in this direction was done, when the amount of bacteria on 3 mm and 5 mm beads were compared. While 3 mm beads have a surface area of 28.274 mm^2^, the surface area of 5 mm beads increases to 78.52 mm^2^. The larger surface provided more colonization space for more bacteria and consequently, higher bacteria counts. The use of 10 mm glass beads in 12 or even 6 well microplates in combination with 5 mL Eppendorf tubes would presumably gain even higher bacteria numbers (not tested in this study). This can be a decisive advantage, when reduction factors of more than 5 log_10_ magnitudes are desired.

Another component that would allow customizing the bead assay to individual needs was the use of polytetrafluoroethylene as bead material. Due to its inert properties, polytetrafluoroethylene is frequently used in the clinic praxis, for example in bypass grafts, shunts or stents [17–19]. The CFU counts of the different beads proved to be invariant with respect to the used material, which suggested a strong influence of available surface area over material properties.

The rotating movement of the shaker during cultivation causes the beads to move. In case of the orbital shaker featuring 25 mm amplitude, the bead quickly circled along the edge of the well. The beads cultivated on an 8 mm shaker, however, performed only small, trembling-like movements. This behavior was expected to cause less shear forces, a more loosely attached biofilm and with that, lower bacteria numbers or a larger standard deviation of the CFU counts. But in fact, both CFU counts proved to be virtually identical. It can be assumed that the mechanical stress caused by the rolling movement between well bottom and bead surface outweighs the influence of the shear forces in the media. The mechanical rolling stress has obviously another consequence, as it contributes to a rather uniform biofilm coverage, which is reflected in the images of the SEM and CLSM.

### Benchmarking the Bead Assay

Since the properties of the bead assay can be positioned somewhere between the MBEC^™^ assay and the CDC biofilm reactor it makes sense to compare selected features of these systems side by side as shown in Table 1.

**Table 1:** Comparison of the bead assay, the MBEC^™^ assay and the CDC biofilm reactor.

	bead assay	MBEC^™^ assay	CDC Biofilm Reactor
availability	any glass bead and microplate selling company	Innovotec Inc.	Bio Surface Technologies Corporation
high throughput capable	limited	yes	no
modular expandable	yes	yes	very limited
varying carrier (coating) materials available	yes—but limited(currently 2 tested)	yes(currently 8 different)	yes(currently 38 different)
up-scaling, down-scaling options	(6,12,24,48 well plates; Ø 2,3,4,5,6,8,10 mm glass beads)	no	no
option to cultivate different species at a time	yes	yes	no
direct carrier handling necessary	yes (tweezers)	no	no
typical CFU/carrier for *P*.*aeruginosa*	7.08–8.03 log10 per bead (Ø 5 mm)	6.1–6.8 log10 per peg [20, 21]	10.8 log10 per coupon[22]
carrier area	78.5 mm^2^ (Ø 5 mm bead)	46.6 mm^2^	127 mm^2^
mean log density (CFU mm^-2^)	5,61 log10 CFU mm^-2^	5.87 log10 CFU mm^-2^[4]	6.5 log10 CFU mm^-2^[22]
shear forces	low	low	high
running costs	low	moderate to high (depending on the coating)	high

The key benefit of the MBEC assay is doubtlessly its true high throughput capabilities while the bead assay has advantages, when low costs are critical or up- and down-scaling of the system is required. The CDC reactor, however, remains irreplaceable wherever high shear forces are necessary, or a wide range of different substrate materials are to be tested.

### Efficacy Testing of Disinfectants

Although the main goal of this study was not to evaluate and compare disinfectants, one demand was, however, to challenge the bead assay in an actual efficacy test. When *P*. *aeruginosa* biofilm was tested against three disinfectants, only peracetic acid and glutaraldehyde were potent enough to accomplish the required 5 log_10_ magnitudes of reduction. In a similar setting, Vikram *et al*. treated 24 h *P*. *aeruginosa* biofilms with 1.25% glutaraldehyde for 10 min and also observed a 5 log_10_ reduction in cell counts [23]. However, when used endoscopes were disinfected according to the standard protocol with a combination of 2% glutaraldehyde and 70% ethanol rinsing followed by forced air-drying, 11 of 60 tested endoscopes still showed positive cultures [24].

The authors of disinfections protocols are mostly well aware of the above mentioned shortcomings and strive for close-to-reality test settings to reflect the situation in a hospital, for instance. One of the still missing steps toward an even more real-life-approach is the incorporation of microbial biofilms to address their higher tolerance against biocides in comparison to planktonic bacteria [5].

The bead assay for biofilms offers an additional platform to the already existing panel of test assays, with which biocides can be tested against biofilms in a reliable, standardized fashion.

## Supporting Information

S1 Flow Chart (PDF)

S1 Spotting Grid (PDF)

## References

[1] Costerton JW, Lewandowski Z, Caldwell DE, Korber DR, Lappin-Scott HM. Microbial biofilms. Annual review of microbiology. 1995;49:711–45. Epub 1995/01/01. 10.1146/annurev.mi.49.100195.003431 . 8561477

[2] Schulte S, Flemming H-C. Ursachen der erhöhten Resistenz von Mikroorganismen in Biofilmen. Chemie Ingenieur Technik. 2006;78(11):1683–9.: 10.1002/cite.200600088

[3] Rutala WA, Weber DJ, (HICPAC) HICPAC. Guideline for Disinfection and Sterilization in Healthcare Facilities, 2008 In: Prevention CfDCa, editor. 2008.

[4] Parker AE, Walker DK, Goeres DM, Allan N, Olson ME, Omar A. Ruggedness and reproducibility of the MBEC biofilm disinfectant efficacy test. Journal of microbiological methods. 2014;102:55–64.: 10.1016/j.mimet.2014.04.013 . 24815513

[5] Tote K, Horemans T, Vanden Berghe D, Maes L, Cos P. Inhibitory effect of biocides on the viable masses and matrices of Staphylococcus aureus and Pseudomonas aeruginosa biofilms. Appl Environ Microbiol. 2010;76(10):3135–42.: 10.1128/AEM.02095-09 20363795 PMC2869147

[6] Allan ND, Omar A, Harding MW, Olson ME. A rapid, high-throughput method for culturing, characterizing and biocide efficacy testing of both planktonic cells and biofilms Science against microbial pathogens: communicating current research and technological advances. Edition: Microbiology Book Series ed: Formatex, Editors: Mendez-Vilas A.; 2011 p. 864–71.

[7] Bardouniotis E, Huddleston W, Ceri H, Olson ME. Characterization of biofilm growth and biocide susceptibility testing of Mycobacterium phlei using the MBEC assay system. FEMS Microbiol Lett. 2001;203(2):263–7. . 11583858 10.1111/j.1574-6968.2001.tb10851.x

[8] Harrison JJ, Stremick CA, Turner RJ, Allan ND, Olson ME, Ceri H. Microtiter susceptibility testing of microbes growing on peg lids: a miniaturized biofilm model for high-throughput screening. Nature protocols. 2010;5(7):1236–54.: 10.1038/nprot.2010.71 . 20595953

[9] ASTM. Standard Test Method for Testing Disinfectant Efficacy against Pseudomonas aeruginosa Biofilm using the MBEC Assay E2799- 12: ASTM international; 2012.

[10] International A. Standard Test Method for Evaluating Disinfectant Efficacy against Pseudomonas aeruginosa Biofilm Grown in CDC Biofilm Reactor using Single Tube Method ASTM: ASTM; 2007 p. 1444–8.

[11] Krankenhaushygiene DGf. BGA Guideline on Testing the Efficacy of Surface Disinfectants. HygMed. 1994;19:477.

[12] DIN EN 14561:2006–08. Chemical disinfectants and antiseptics—Quantitative carrier test for the evaluation of bactericidal activity for instruments used in the medical area—Test method and requirements (phase 2, step 2); German version EN 14561:2006.

[13] DIN EN 13727:2014–03. Chemical disinfectants and antiseptics—Quantitative suspension test for the evaluation of bactericidal activity in the medical area—Test method and requirements (phase 2, step 1); German version EN 13727:2014

[14] Cholodny N. Zur Morphologie der Eisenbakterien Gallionella und Spirophyllum. Berichte der Deutschen Botanischen Gesellschaft. 1924;XLII(2):35–44.

[15] Webster P, Wu S, Gomez G, Apicella M, Plaut AG, St Geme JW 3rd. Distribution of bacterial proteins in biofilms formed by non-typeable Haemophilus influenzae. J Histochem Cytochem. 2006;54(7):829–42.: 10.1369/jhc.6A6922.2006 . 16549506

[16] Liu Y, Tay JH. The essential role of hydrodynamic shear force in the formation of biofilm and granular sludge. Water Res. 2002;36(7):1653–65. . 12044065 10.1016/s0043-1354(01)00379-7

[17] Barman AA, Batiuchok W, Chaudry SS, Creedon JJ. Use of interposed polytetrafluorethylene (PTFE) graft in distal splenorenal shunt. Cardiovasc Dis. 1981;8(4):555–7. 15216183 PMC288001

[18] Loh A, Chester JF, Taylor RS. PTFE bypass grafting to isolated popliteal segments in critical limb ischaemia. Eur J Vasc Surg. 1993;7(1):26–30. . 8454073 10.1016/s0950-821x(05)80539-0

[19] Angeloni S, Merli M, Salvatori FM, De Santis A, Fanelli F, Pepino D, et al Polytetrafluoroethylene-covered stent grafts for TIPS procedure: 1-year patency and clinical results. Am J Gastroenterol. 2004;99(2):280–5. . 15046218 10.1111/j.1572-0241.2004.04056.x

[20] Harrison JJ, Turner RJ, Ceri H. High-throughput metal susceptibility testing of microbial biofilms. BMC Microbiol. 2005;5:53: 10.1186/1471-2180-5-53 16202124 PMC1262724

[21] Carson L, Chau PKW, Earle MJ, Gilea MA, Gilmore BF, Gorman SP, et al Antibiofilm activities of 1-alkyl-3-methylimidazolium chloride ionic liquids. Green Chemistry. 2009;11(4):492–7.: 10.1039/B821842K

[22] Buckingham-Meyer K, Goeres DM, Hamilton MA. Comparative evaluation of biofilm disinfectant efficacy tests. Journal of microbiological methods. 2007;70(2):236–44. Epub 2007/05/26.: 10.1016/j.mimet.2007.04.010 . 17524505

[23] Vikram A, Bomberger JM, Bibby KJ. Efflux as a glutaraldehyde resistance mechanism in Pseudomonas fluorescens and Pseudomonas aeruginosa biofilms. Antimicrob Agents Chemother. 2015;59(6):3433–40.: 10.1128/AAC.05152-14 25824217 PMC4432172

[24] Fraser VJ, Zuckerman G, Clouse RE, O'Rourke S, Jones M, Klasner J, et al A prospective randomized trial comparing manual and automated endoscope disinfection methods. Infect Control Hosp Epidemiol. 1993;14(7):383–9. . 8354869 10.1086/646766

