# Supplementary figures and images for: The Bead Assay for Biofilms: A Quick, Easy and Robust Method for Testing Disinfectants
Source: PLoS One. 2016 Jun 17;11(6):e0157663. doi: 10.1371/journal.pone.0157663 (PMC4912112; doi:10.1371/journal.pone.0157663)

# BEAD ASSAY FOR BIOFILMS

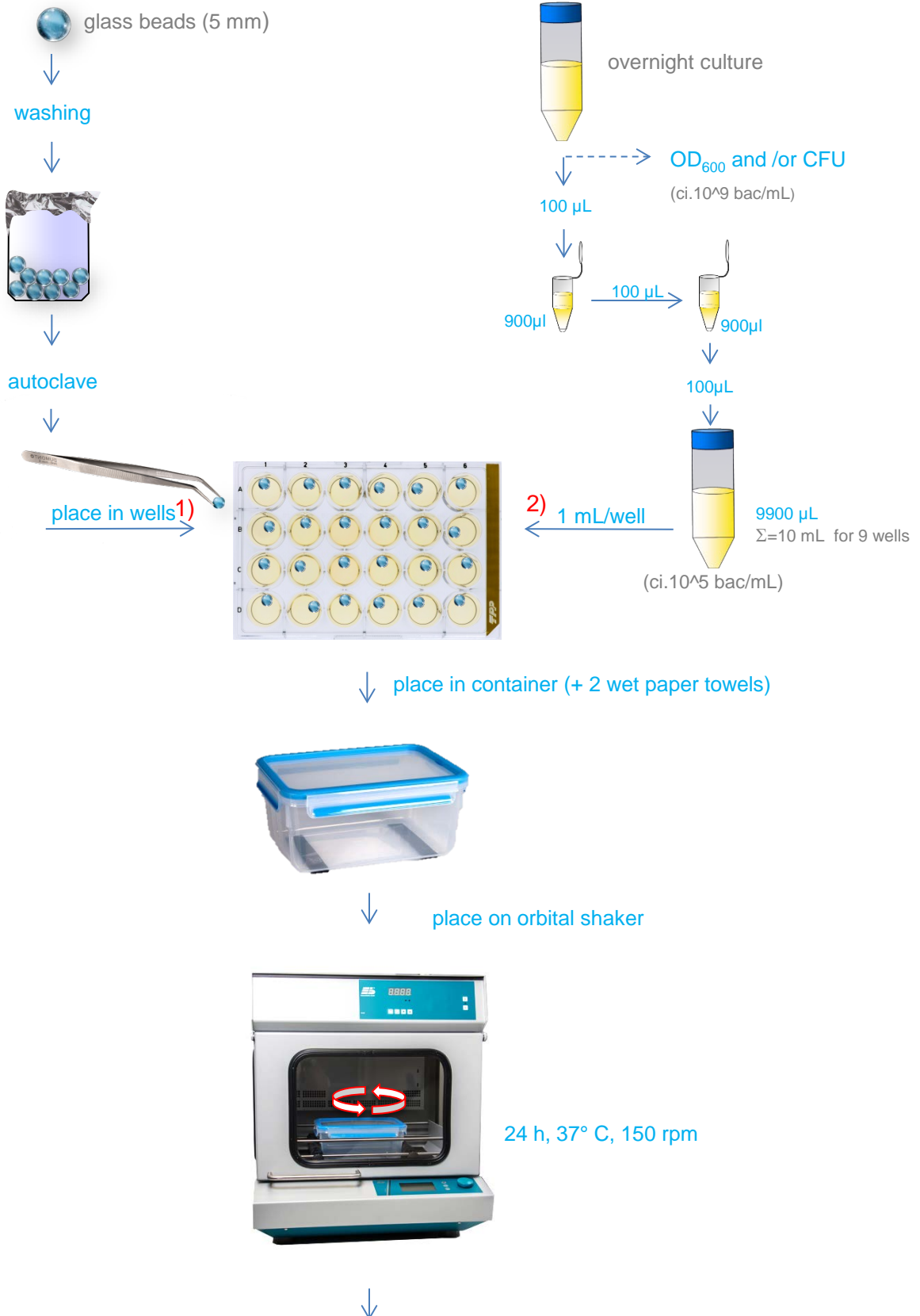

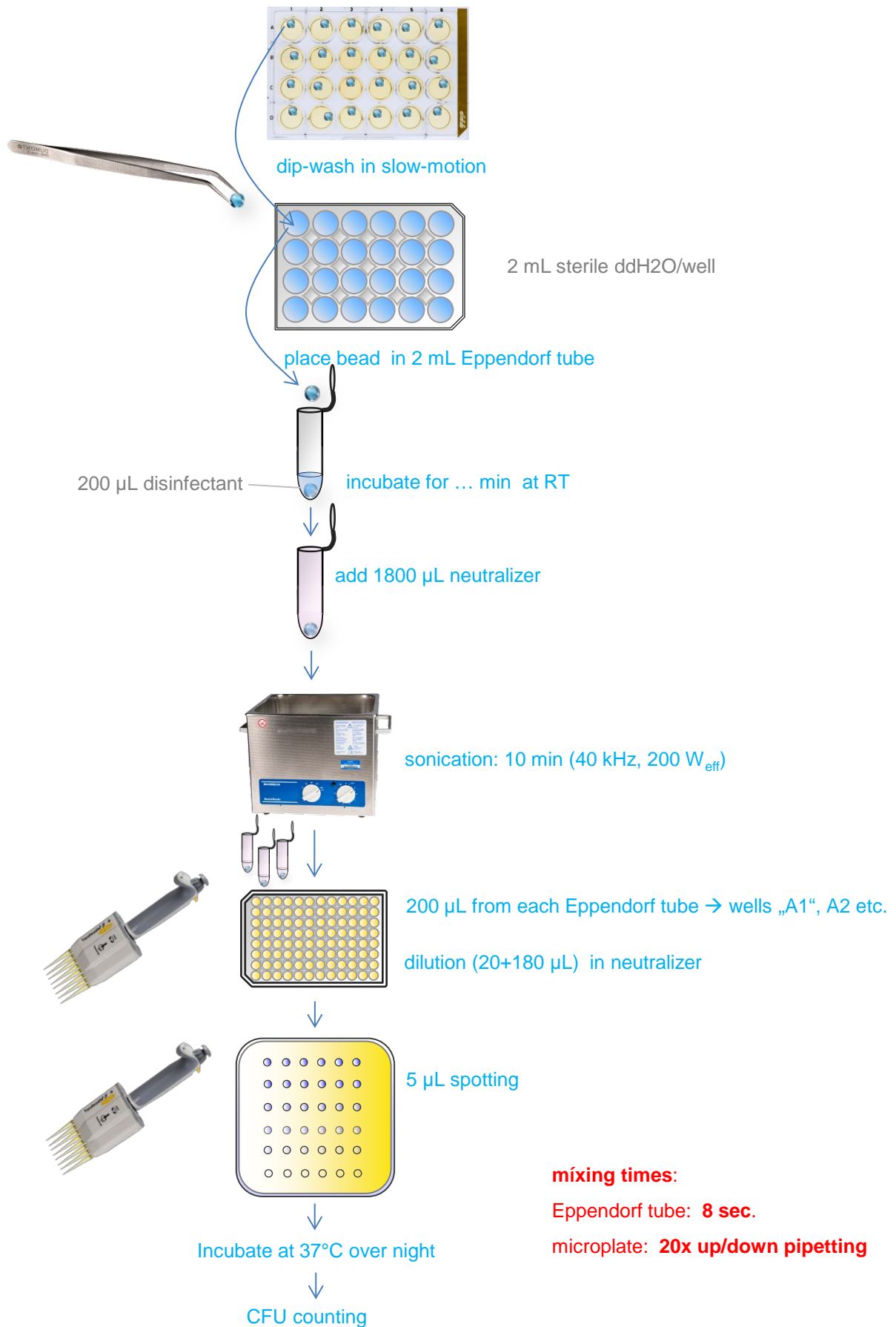

Supplement: S1 Flow Chart — (PDF) [file pone.0157663.s001.pdf]
